# The impact of climate change on plant diversity in peatlands in Sichuan province, China

**DOI:** 10.3389/fpls.2026.1762128

**Published:** 2026-04-17

**Authors:** Zhengxuan Wei, Yuelin Wang, Wende Chen, Jie Ran, Zhijia Wang, Ruojing Chen

**Affiliations:** College of Geography and Planning, Chengdu University of Technology, Chengdu, China

**Keywords:** climate scenarios, MaxEnt model, peatland plants, Sichuan province, species richness

## Abstract

**Introduction:**

There are extensive peatland habitats in Sichuan Province, a crucial ecological barrier in southwest China. However, climate change has caused widespread degradation of peatland habitats, creating significant survival challenges for many peatland species.

**Methods:**

Using the MaxEnt model, we can assess the link between species distribution and environmental variables, forecast the geographical patterns of significant peatland plant richness hotspots in Sichuan Province from 1981 to 2010, and identify significant environmental driving factors. The probable distribution patterns for major peatland plants are further forecasted by combining three future(2071-2100s) climate outlooks, corresponding to SSP1-2.6 (low), SSP3-7.0 (medium), and SSP5-8.5 (high) emission trajectories.

**Results:**

The foremost variables determining the geographic distribution of peatland plants in Sichuan Province include SCD (Snow cover days, 41.5%), Slope (40.4%), TWI (Topographic wetness index, 7.3%), Bio15 (Precipitation seasonality, 2.2%), and Bio3 (Isothermality, 2.1%). SCD (optimal range of 120–240 days) plays a key role in regulating soil temperature, humidity, and protecting plants from cold, significantly affecting peatland plants in Sichuan Province. Overall, the distribution of 50 prominent peatland plants reveals a spatial pattern with northwest high and southeast low, with Zoige County having the richest species diversity. As climate conditions evolve, the possible habitats of major peatland plants will generally decrease, species richness will diminish, and biodiversity shifts near the boundaries of plateaus and basins will be particularly substantial. The majority of the advantageous peatland plant distribution regions have been included in the protection system. However, some potentially suitable and shrinking areas that have not been covered, and they should be prioritized in developing the protection network.

## 1 Introduction

Peatland plants play a crucial role in carbon storage, water quality regulation, and biodiversity maintenance (Liu et al., 2022; Laine et al., 2022). Although climate change may lead to the expansion of peatlands in certain regions, its negative impacts are more significant (Crichton et al., 2025). Climate change has exacerbated the drying and organic carbon decomposition of peatlands, increasing the risk of drought and wildfires, transforming peatlands from carbon sinks to carbon sources, and leading to severe degradation of peatlands (Loisel and Gallego-Sala, 2022; Wilkinson et al., 2023). At present, the global peatland area has decreased by over 10% (Leifeld and Menichetti, 2018), accounting for only 3% of the global land area (Günther et al., 2020). These changes not only affect the peatland ecosystem but also pose a significant threat to biodiversity (Yuan et al., 2024). Given the increasing degradation of peatlands, understanding the impact of climate change on peatland plant diversity is an urgent research priority.

Species diversity is the core of ecosystem function (Albrecht et al., 2021). In addition to promoting the diversification of ecological functions and improving ecosystems’ adaptation, it also greatly enhances the ecosystem’s ability to resist biodiversity loss by providing functional redundancy, improving adaptation, promoting interspecies assistance, and raising resource use efficiency (Token et al., 2022; Yu et al., 2024). Currently, researchers use methods such as MaxEnt, RF, GAM, CTA, GBM, and GLM to simulate situations where species may adapt under various environmental conditions, all of which can efficiently capture the nonlinear relationship between species and environment, and have good generalization ability and prediction accuracy (Cho et al., 2022; Wani et al., 2025). However, compared with other species distribution models, the MaxEnt model performs well even with incomplete distribution data, since it only requires species presence data for model construction (Ahmadi et al., 2023). Additionally, it can accept both continuous and categorical variables as predictors (Zhao et al., 2022). Previous studies have successfully predicted the potential distribution of various species using the MaxEnt model, such as *Oxalis debilis* Kunth (Qin and Li, 2023), *Erigeron canadensis* L (Yan et al., 2020), and *Ulex europaeus* (Christina et al., 2020). This method has also been used to simultaneously study multiple plants, such as the *Amaranthaceae* family (Lin et al., 2025) and the *Bidens genus* (Xiao et al., 2025). Although the above research has made significant progress in predicting the potential distribution of species, it is primarily focused on the distribution of one or more species within specific genera or families. Many scholars tend to use this model to predict the potential distribution of endangered or invasive species, without considering multiple plants from different families or genera, which limits its applicability in predicting changes in biodiversity. Based on this, this study collected 50 different families or genera of peatland plants in Sichuan Province and used the MaxEnt model to predict the impact of climate change on their distribution, while further exploring changes in diversity to enrich the research content in this field.

This study applies the MaxEnt model, incorporating occurrence records of 50 peatland plants in Sichuan Province and environmental variables (climate, terrain, soil), to estimate the potential impacts of climate change on the plant diversity patterns of 50 dominating peatlands in Sichuan Province. The specific objectives are: ① Determine the principal environmental factors shaping the distribution of peatland plants within Sichuan Province; ② Construct a spatial pattern of plant richness in dominant peatlands for the current year (1981-2010), further predict the trend of species richness changes towards the end of the 21st century, and identify regions with relatively stable biodiversity; ③ Analyze the gaps in existing protected areas and propose suggestions for optimizing peatland and key species conservation networks. We expect that the research findings will be able to offer an analytical foundation for the preservation and restoration of peatlands in Sichuan Province.

## 2 Materials and methods

### 2.1 General situation

**Figure 1:**
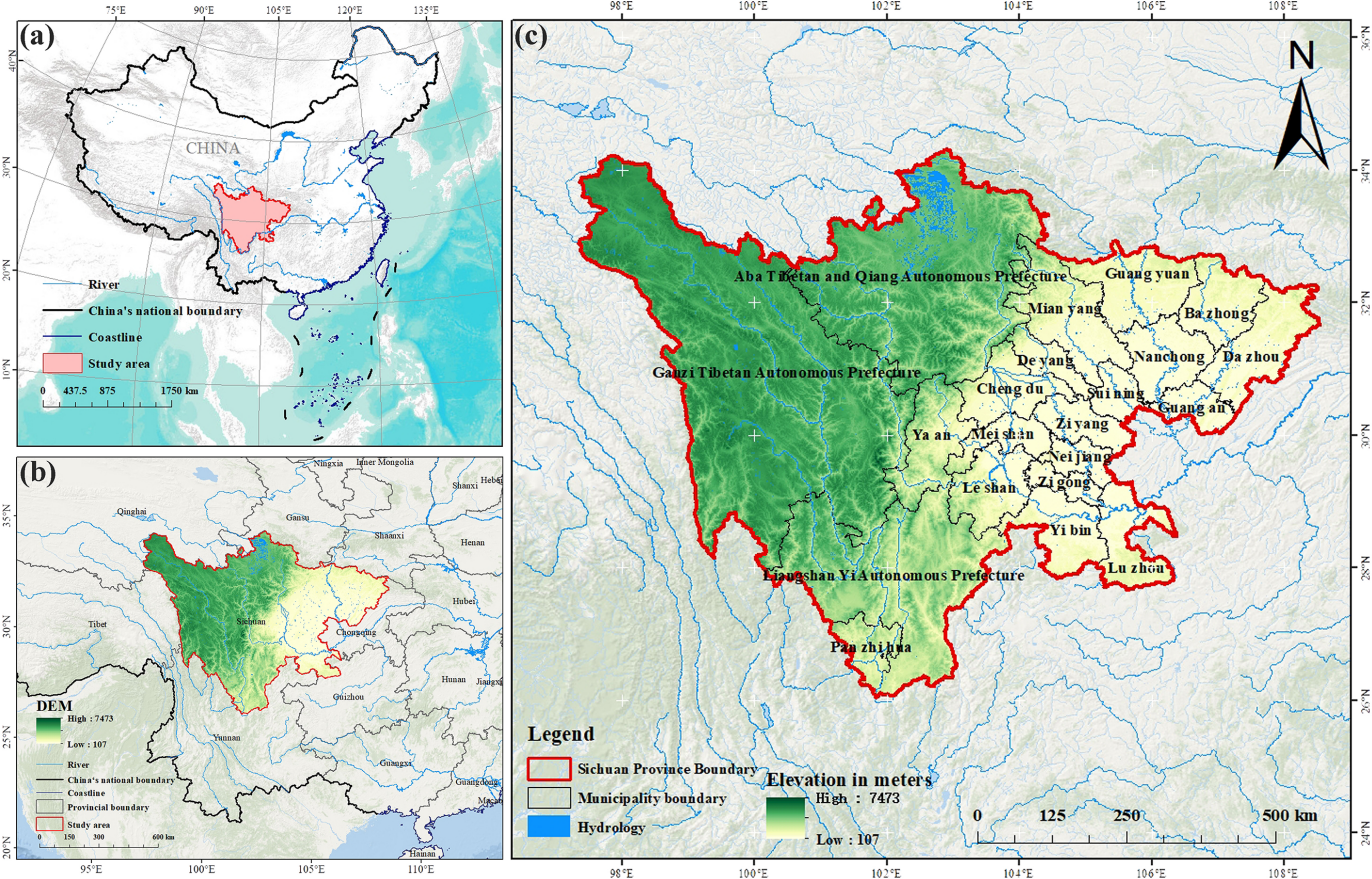
Research area: Sichuan Province. **(a)** Location in China; **(b)** Location in Southwest China; **(c)** Administrative divisions of Sichuan Province.

Situated in southwest China, Sichuan Province occupies an area of 48.61×10^4^ km². It serves as a crucial transitional area between the central and lower Yangtze River plain and the Qinghai–Tibet Plateau (**Figure 1**). Within the province, there is a wide range of altitudes and diverse landforms. The westernregion encompasses the Qinghai–Tibet Plateau and the Hengduan Range, the southern part borders the Yunnan-Guizhou Plateau, the central and eastern areas consist of the Sichuan Basin, and the northeastern region is occupied by the Qinba Mountains (Liu et al., 2022). Sichuan Province has a large span of peatlands, covering multiple plateaus and mountainous areas (Peng et al., 2021). The 50 plant species selected in this study are representative of peatlands in western Sichuan, covering 18 families and 27 genera (**Supplementary Table 1**). These plant species span hydrological niches from aquatic to mesophytic, thus exhibiting distinct hydrological niche adaptations, with their distribution data derived from the findings of the “Comprehensive Survey of Chinese Marsh Wetland Resources and Their Main Ecological Environment Benefits” (https://www.geodata.cn/data/datadetails.html?dataguid=177510600546953&docId=12078%20%5b2022-08-10%5d), and the selected species represent the top 50 most widely distributed peatland plants in Sichuan Province. We utilized the SpThin package in R language to set the minimum distance between sample points to 1 kilometer (Aiello-Lammens et al., 2015), and a total of 1439 distribution points of 50 dominant peatland plants were obtained for model construction to ensure the uniformity of sample distribution and reduce the impact of bias on the data.

### 2.2 Selection of environmental factors

To simulate the MaxEnt model, this study chose 28 environmental variables in total, consisting of 20 bioclimatic variables, 4 terrain factors, and 4 soil parameters (**Table 1**). These bioclimatic variables are derived from relevant climate parameters in the CHELSA database (https://chelsa-climate.org), which has a geographical resolution of 0.0083 degrees and contains 19 bioclimatic variables (Bio1-Bio19) and SCD (snow cover days) to fully depict the climatic conditions of the research region. This study adopts three representative pathways (SSP126, SSP370, and SSP585) to reflect patterns of social progress and emission intensities, thereby simulating climate change trends across low, medium, and high-emission futures. The average value of the five models of GFDL-ESM4 (USA), UKESM1-0-LL (UK), MPI-ESM1-2-HR (Germany), IPSL-CM6A-LR (France), and MRI-ESM2-0 (Japan) was simultaneously determined using the map algebra tool in GIS10.8.1, to mitigate systematic biases of single models and enhance the reliability and representativeness of future climate projections. Elevation data is obtained through the geographic spatial data cloud (https://www.gscloud.cn/). It provides a digital elevation model (DEM) with a resolution of 30 meters. On this basis, the terrain analysis tool of ArcGIS 10.8.1 software is used to process the DEM, extract slope and aspect information to reflect the geomorphic features of the target area. The soil data originates from the SoilGrids database (https://soilgrids.org/). We selected soil carbon content, nitrogen content, pH value, and bulk density indicators within the depth ranges of 0–5 cm and 5–15 cm. To more accurately reflect the characteristics of the surface soil, the data from two depth layers were averaged. LULC data were extracted from the most recent version (V6) of the China Land Cover Dynamic Dataset (https://zenodo.org/record/4417810), while information on nature reserves was obtained from the Resources and Environmental Sciences Data Center of the Chinese Academy of Sciences (https://www.resdc.cn/data.aspx?DATAID=272).

**Table 1:** 28 types of environmental variables.

Type	Variable	Variable description	Unit
Bioclimatic Variables	Bio1	Average Annual Temperature	°C
	Bio2	Average Daily Temperature Variation	–
	Bio3	Isothermality	°C
	Bio4	Temperature Seasonality	°C
	Bio5	Highest Temperature of Hottest Month	°C
	Bio6	Lowest Temperature of Coldest Month	°C
	Bio7	Annual Temperature Difference	°C
	Bio8	Average Temperature During Wettest Quarter	°C
	Bio9	Mean Temperature of the Dryest Period	°C
	Bio10	Average Temperature During Hottest Quarter	°C
	Bio11	Average Temperature During Coldest Quarter	°C
	Bio12	Yearly Precipitation	mm
	Bio13	Precipitation During the Wettest Month	mm
	Bio14	Precipitation During the Driest Month	mm
	Bio15	Seasonal Precipitation Variability	mm
	Bio16	Rainfall in the Wettest Quarter	mm
	Bio17	Rainfall in the Driest Quarter	mm
	Bio18	Rainfall in the Warmest Quarter	mm
	Bio19	Rainfall in the Coldest Quarter	mm
	SCD	Snow Cover Days	d
Terrain Variables	ASP	Aspect	°
	Dem	Elevation	m
	SLO	Slope	°
	TWI	Topographic Wetness Index	–
Soil Variables	BD	Bulk Density	g/cm³
	OC	Organic Carbon	g/cm³
	PH	Soil pH	–
	TN	Total Nitrogen	g/cm³

Afterwards, to reduce the high correlation among environmental variables on model construction, a Pearson correlation examination was executed on 28 ecological variables, with pairs showing a correlation coefficient whose absolute value exceeds 0.75 considered highly collinear (**Supplementary Table 2**). Priority is given to retaining variables with clearer ecological significance and higher contribution and substitution importance in the preliminary modeling, and the rest variables are excluded (Zhang et al., 2024). After screening, 10 key variables were ultimately selected for subsequent species distribution simulations (**Table 2**). Additionally, the WGS-1984 geographic coordinate system is employed, and boundary ranges are consistently specified for all variable layers using a spatial accuracy of 1km×1km so as to guarantee the consistency of geographical data.

**Table 2:** Environmental indicator on the distribution of dominant peatland plant species in Sichuan.

Variable	Variable description	Unit	Percent contribution	Permutation importance
Bio3	Isothermality	–	2.1	0.6
Bio4	Temperature Seasonality	°C	2.0	2.9
Bio13	Precipitation During the Wettest Month	mm	1.1	3.2
Bio14	Precipitation During the Driest Month	mm	1.1	3.3
Bio15	Seasonal Precipitation Variability	mm	2.2	5.2
SCD	Snow Cover Days	d	41.5	43.5
PH	Soil pH	–	1	2.2
TWI	Topographic Wetness Index	–	7.3	4.5
Asp	Aspect	(°)	1.4	1.1
Slo	Slope	(°)	40.4	28.7

### 2.3 Modeling species habitat distribution

The possible distribution zones of peatland plants were predicted in this study using MaxEnt. The algorithm just needs environmental data and species distribution data to produce extremely accurate prediction results (Castiello et al., 2025). In this study, the MaxEnt model was optimized with the ENMeval package, and RM = 2 with FC = LQH was selected as the optimal parameter combination (**Supplementary Figure 1**), while 25% of the occurrence data were randomly selected as test data and the remaining 75% were used for model training. The number of replicates was set to 10, and the maximum number of background points was set to 10,000. A species distribution model was constructed using the Jackknife Test method, and the distribution probability of the species was represented in the form of logistic output for subsequent visualization analysis. The default values of other parameters were retained (Hill et al., 2024). In the simulation results, a higher value for the area under the curve (AUC) indicates more reliable model predictions. The model demonstrates excellent precision when the AUC exceeds 0.9, while predictions are relatively trustworthy when the value falls between 0.7 and 0.9 (Ahmed et al., 2021). After 10 repeated modeling attempts, the average result is taken to reduce random errors. And the species existence probability logic value P, which ranges from 0 to 1, was acquired in ArcGIS 10.8.1. The likelihood of the species existing there is higher when the value is closer to 1 (Qin et al., 2017; Robeck et al., 2024). Simultaneously, the suitable distribution region was separated into two categories (**Supplementary Figure 2**), namely suitable habitat (P≥0.5) and non-suitable habitat (P<0.5), with 0.5 serving as the threshold (Scherrer et al., 2020; Chi et al., 2023; Wang et al., 2025).

## 3 Results

### 3.1 Performance and validation of the model

The distribution of 50 prominent plants in peatlands in Sichuan Province was successfully simulated in this study, with an average AUC value of 0.9287. Among them, 46 species had AUC values exceeding 0.9. Only four species have AUC values between 0.8 and 0.9, meaning that their model prediction results demonstrate considerable reliability (**Supplementary Table 3**).

### 3.2 Important factors affecting the distribution of peatland plants in Sichuan

**Figure 2:**
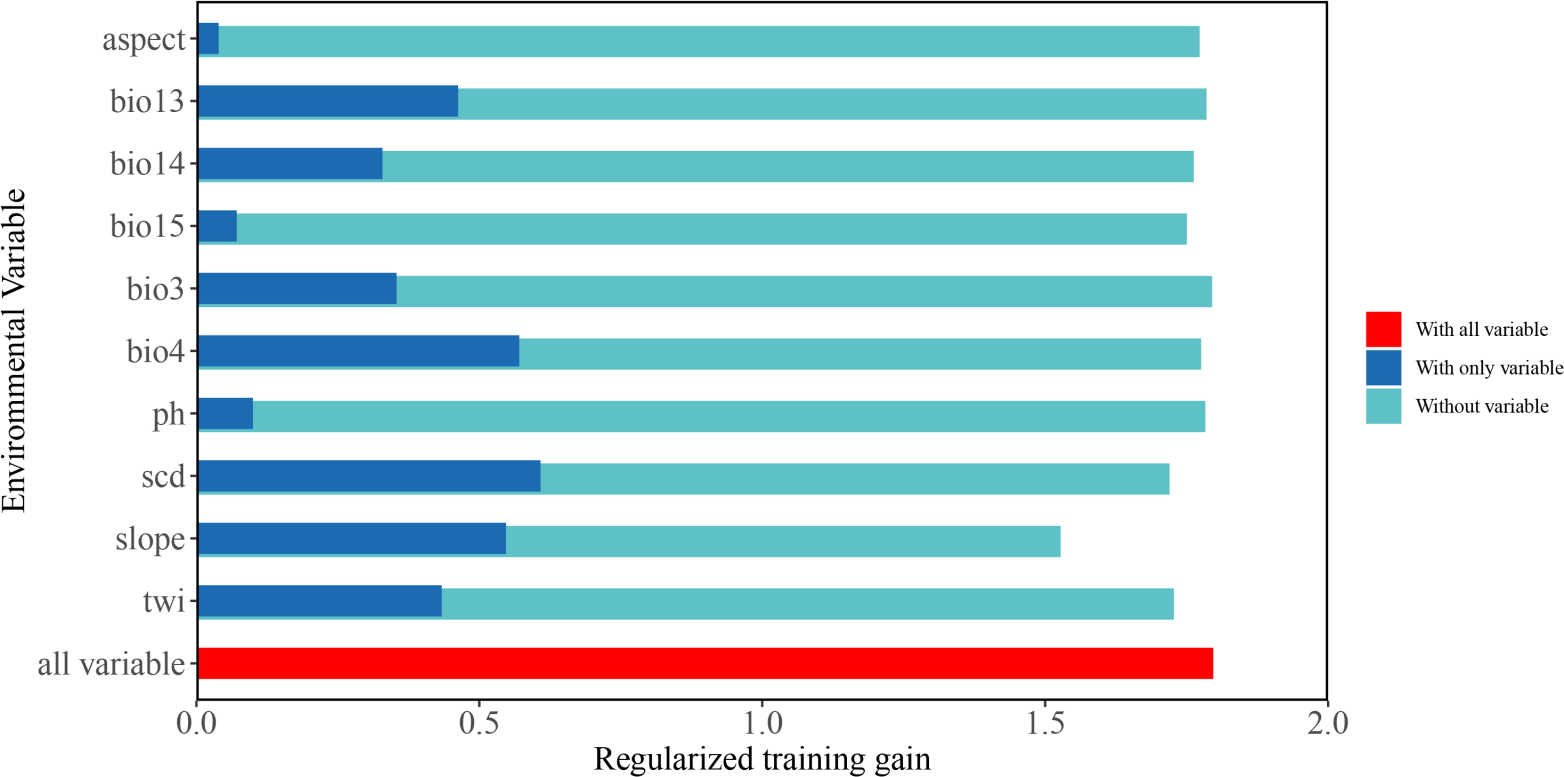
Jackknife test results.

**Table 2** shows the impact of various environmental variables on the potential habitat of peatland plants in Sichuan Province. Among them, the five factors that have the most effect on habitat appropriateness are SCD (Snow cover days, 41.5%), Slo (Slope, 40.4%), TWI (Topographic wetness index, 7.3%), Bio15 (Precipitation seasonality, 2.2%), and Bio3 (Isothermality, 2.1%), together making up more than 80% of the total influence. Among these factors, SCD (41.5%) and Slo (40.4%) have high replacement importance. The knife analysis method (**Figure 2**) shows the relative importance of a single environmental factor (blue bar), the remaining variables after removing the factor (cyan bar), and the complete model containing all variables (red bar). Therefore, when using SCD, Slo, and Bio15, they contribute significantly to predicting the suitability of peatland plant habitats.

**Figure 3:**
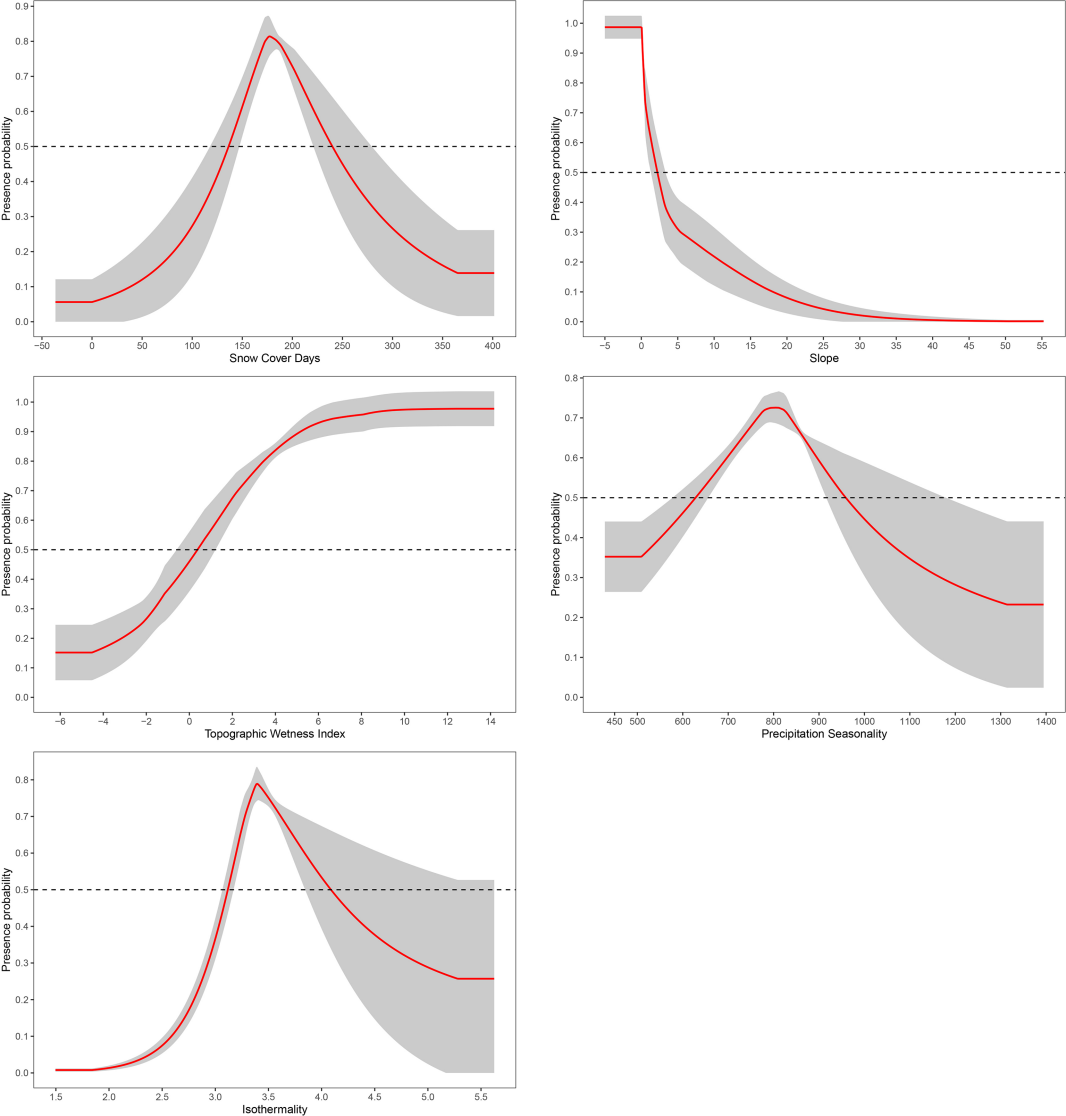
Response curve for significant environmental factors.

The response curve demonstrates how important the environmental factors and plant adaptability are to each other in Sichuan Province’s peatlands (**Figure 3**). This study classified the variables with the highest contribution as the top factors influencing the latent distributions of the peatland plants of Sichuan Province and analyzed the response curves for the main factors affecting each species. In **Figure 3**, the average and standard deviation of ten model runs are shown by the red curve and grey shadow, respectively. In Sichuan Province, there is a definite unimodal relationship between the likelihood of peatland plant occurrence and SCD. The best range for SCD is 120 days to 230 days, with 180 days having the highest probability (approximately 0.83). The relationship between peatland plants and slope in Sichuan Province shows a monotonic decrease, and the probability of existence sharply decreases with increasing slope. The optimal range of slope is between 0° and 2.5°, with the highest probability (approximately 0.98) occurring at 0°. The probability of plant existence in peatlands in Sichuan Province gradually increases with the increase of TWI. When TWI is greater than 0.2, its probability of existence is above 0.5, and the existence rate (about 0.97) reaches its highest point when TWI is 10. The peatland plants in Sichuan Province also exhibit a clear unimodal relationship with Bio15 and Bio3. The suitable range for Bio15 is 600mm to 900mm, and when Bio15 reaches 800mm, there is a probability of reaching its peak (approximately 0.72). The optimal scope for Bio3 is between 3.3° and 4.2°, and the probability of existence reaches its highest value (approximately 0.78) when Bio3 reaches around 3.4°.

### 3.3 Changes in plant richness of peatlands in Sichuan province under different climate scenarios

**Figure 4:**
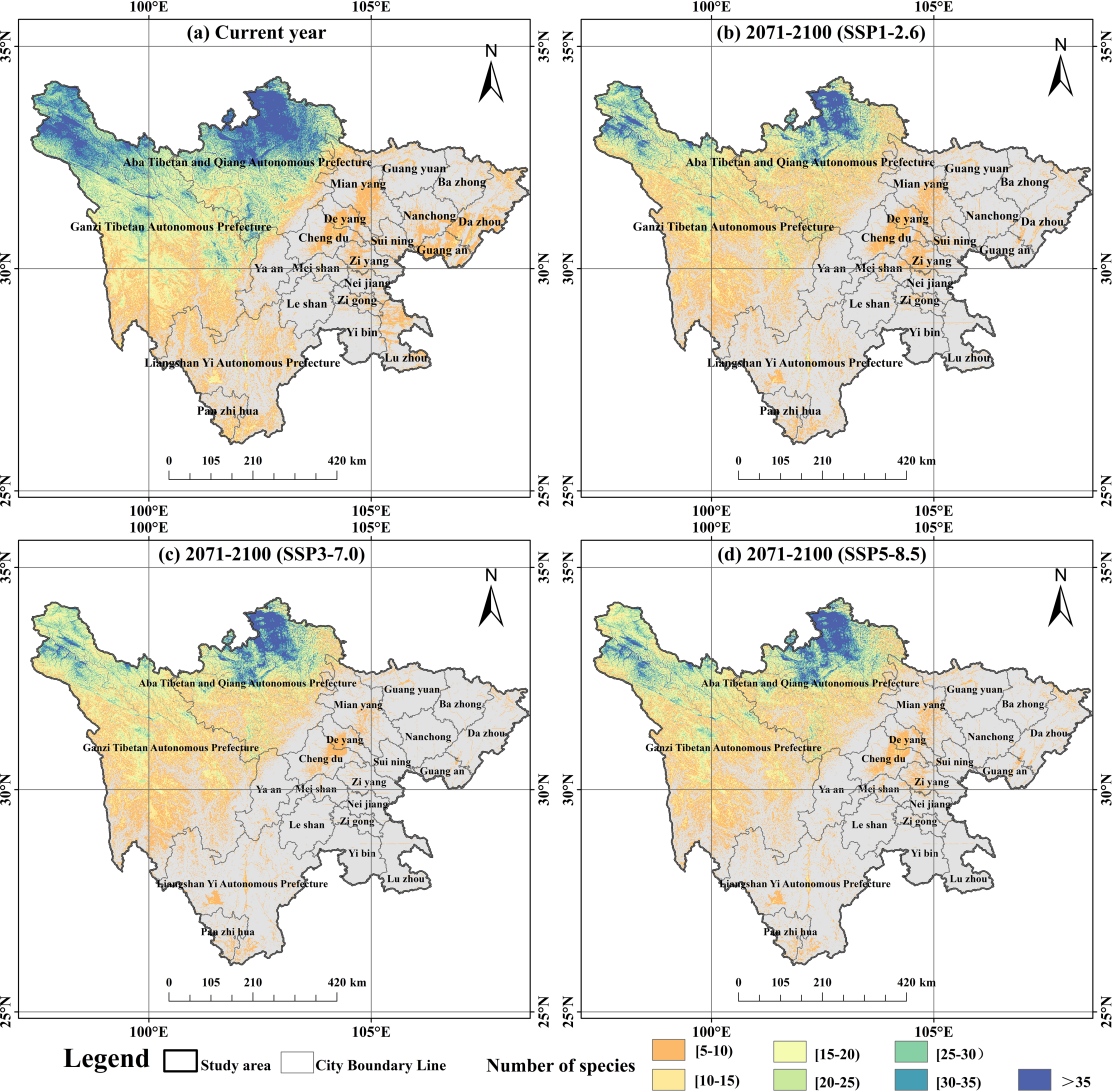
Distribution map of species richness hotspots of dominant peatland plants in Sichuan Province under different scenarios: **(a)** current year; **(b)** 2071–2100 (SSP1–2.6); **(c)** 2071–2100 (SSP3–7.0); **(d)** 2071–2100 (SSP5–8.5).

After excluding farmland coverage areas, this study stacked the distribution of 50 dominant peatland plants and referred to existing research to define the top 25% of species richness as species richness hotspots. Based on this, species richness hotspot maps were drawn for different scenarios (**Figure 4**). The model’s simulation outcomes show that the potential distribution of peatland plants in Sichuan Province gradually decreases from northwest to southeast. Specifically, Sichuan Province contains more than 30 types of peatland plants concentrated in the high-altitude transition from the western Sichuan Plateau to the basin (such as the middle and southern regions of Aba Prefecture and the northeastern area of Garze Prefecture), with a total area of 5.06×10^4^ km², constituting 10.41% of the province’s overall area. The areas with species richness between 5 and 10 (8.05×10^4^ km², 16.56%) are mainly concentrated in the core areas of cities such as Chengdu, Deyang, Mianyang, Nanchong, and Dazhou. The areas with species richness between 10 and 20 (8.35×10^4^ km², 17.18%) are mainly located in the southern part of Garze Prefecture, the central southern part of Liangshan Prefecture, and the hilly areas around the basin. The area with species richness between 20 and 30 (6.35×10^4^ km², 13.06%) covers the central part of Garze Prefecture, the northern part of Liangshan Yi Autonomous Prefecture, and the western edges of Ya’an and Meishan. Finally, peatland plant hotspots with species richness above 35 (2.14×10^4^ km², 4.41%) are concentrated in local areas of Ruoergai County in Aba Prefecture and Shiqu County in Garze Prefecture.

**Figure 5:**
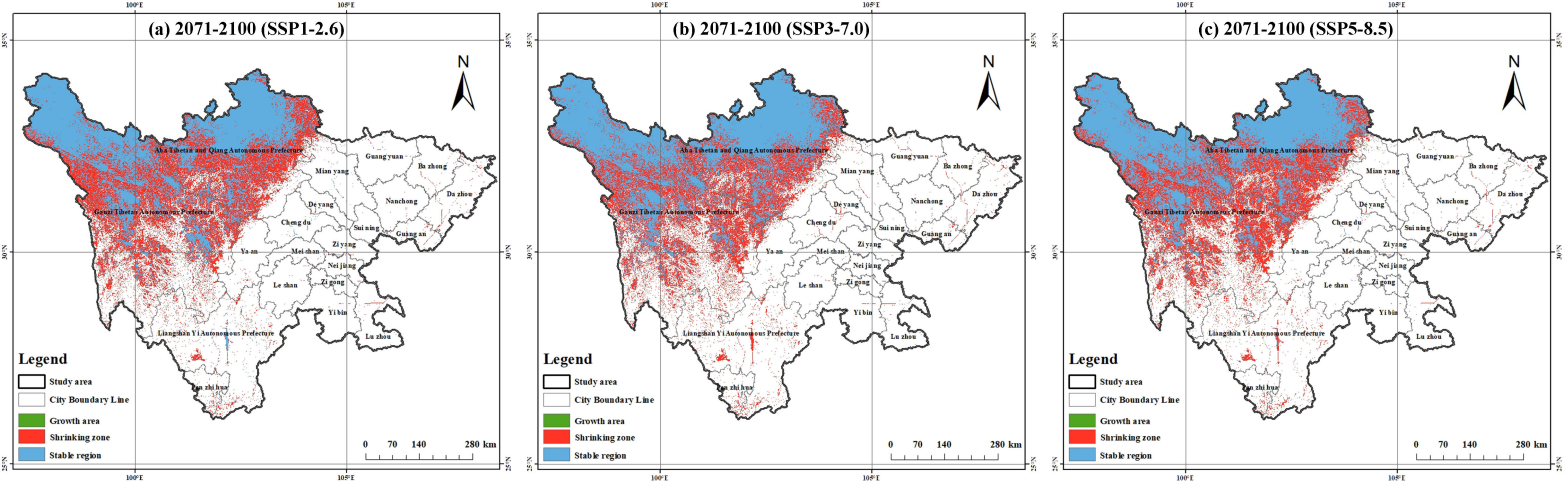
Prediction of species richness of dominant peatland plant species: **(a)** 2071-2100 (SSP1–2.6); **(b)** 2071-2100 (SSP3–7.0); **(c)** 2071-2100 (SSP5–8.5).

**Figure 6:**
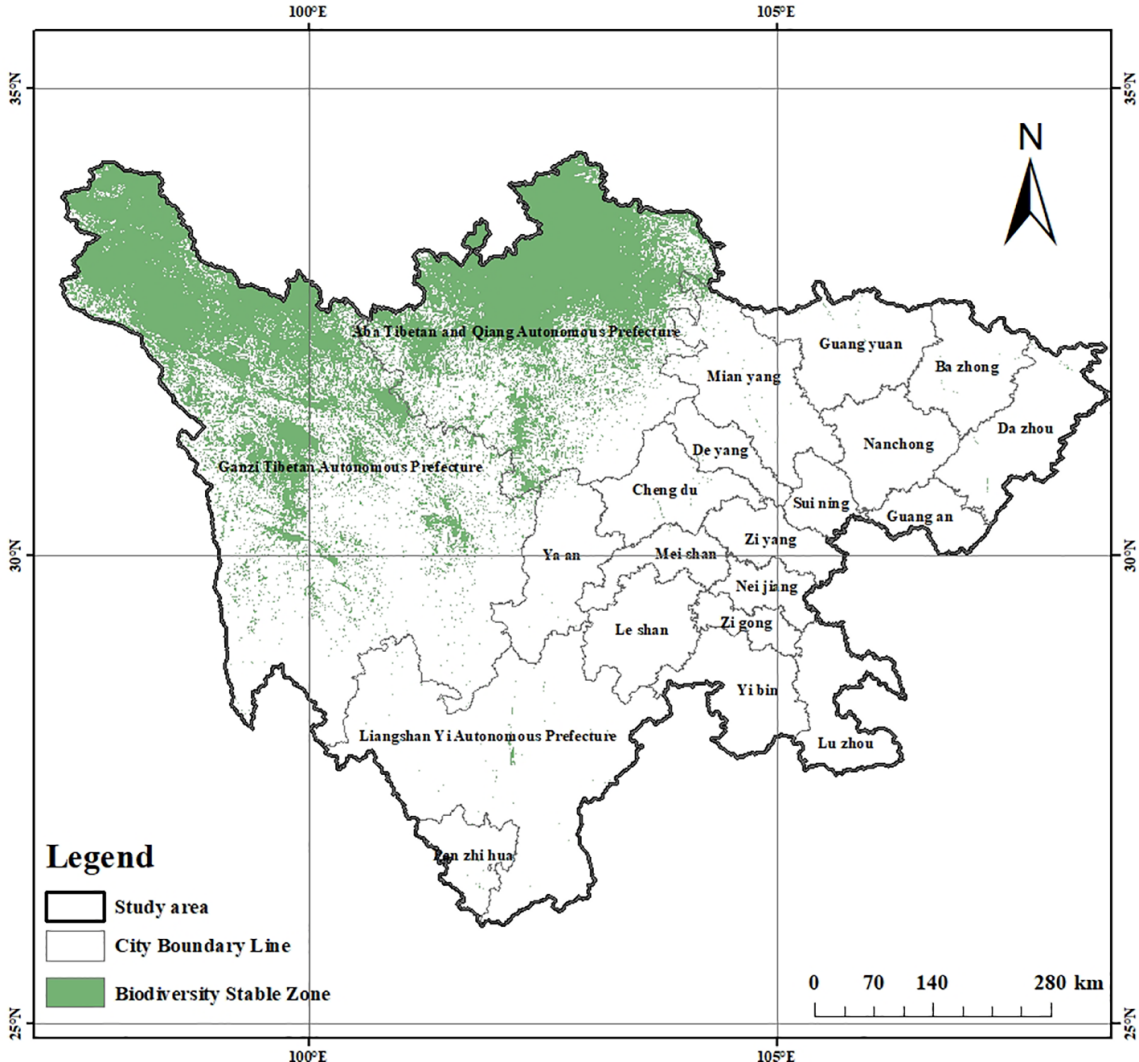
Biodiversity stable zone.

This study plotted the variations in plant richness in Sichuan peatlands based on the model prediction results (**Figure 5**). To better illustrate how future climate change will affect richness, we divided regional changes into three categories: shrinking regions, expanding regions, and stabilizing regions. Among these, the shrinking area is the area where future richness is comparatively lower than current richness; the growth zone is the area where future richness will rise in comparison to current levels; and the stable zone is an area where richness is neither changing now nor in the future. By the end of the 2100s, the area of species richness between 5 and 15 in the SSP126 climate scenario expanded. In addition, under the SSP370 climate scenario, the area of regions with species richness between 10 and 15 has also increased (**Supplementary Table 4**). However, in the remaining cases, the area of species richness showed varying degrees of reduction, and the reduced area was much greater than the increased area. These areas continue to be stable biodiversity hotspots in a variety of temporal and spatial contexts, as shown by the overlap between the current annual richness hotspots and the richness hotspots under three climate scenarios at the end of the 21st century (**Figure 6**). From the distribution of stable areas, most of them are located in Ruoergai County and Shiqu County.

### 3.4 Changes in the spatial pattern of peatland plants in Sichuan province under different climate scenarios

This study also plotted the potential habitat suitability distribution of 50 dominant plants in Sichuan’s peatlands based on the present and the future three types of climate change (2071-2100), along with a comparison of shifts in dominant plant appropriate regions under climate variables.

**Figure 7:**
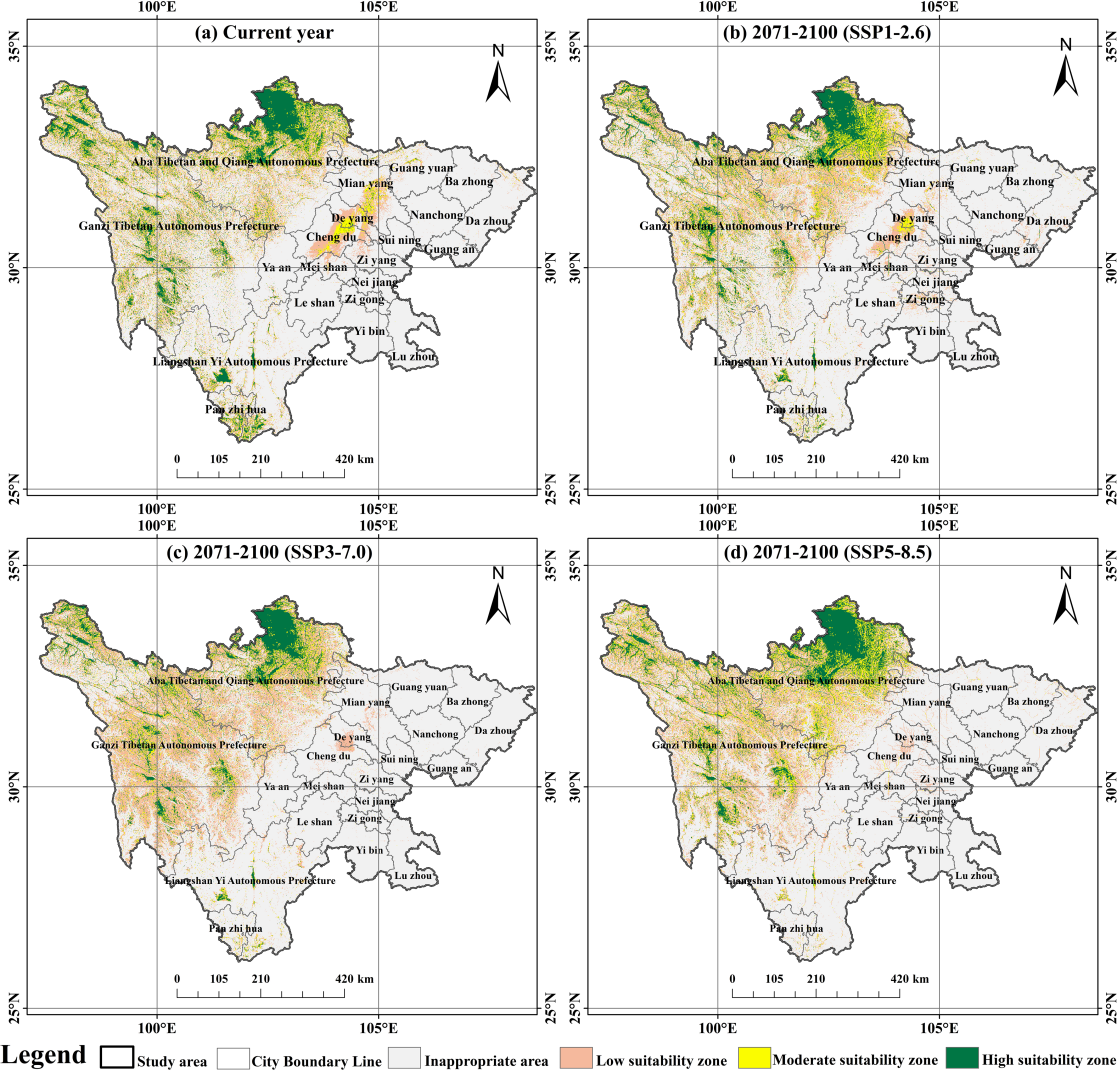
Potential suitable habitats for 50 peatland plants in Sichuan Province under different climate scenarios: **(a)** Current year; **(b)** 2071–2100 (SSP1–2.6); **(c)** 2071–2100 (SSP3–7.0); **(d)** 2071–2100 (SSP5–8.5). Suitability: green = high (HSI > 0.6), yellow = medium (0.4–0.6), light orange = low (0.2–0.4).

As shown in **Figure 7a**, the current potential distribution of peatland plants in Sichuan Province is higher in the western region than in the eastern region. The high suitability zone (3.71×10^4^ km², 7.63%) is primarily found in the southern portion of Ganzi Prefecture, the northern portion of Aba Prefecture, and the southern portion of Liangshan Prefecture. The moderate suitability zone (3.45×10^4^ km², 7.10%) mainly covers local areas of Chengdu, Deyang, and Mianyang. The low suitability zone (8.00×10^4^ km², 16.46%) is mainly located on the periphery of the Chengdu Plain, including areas such as Deyang, Meishan, and Mianyang, and is a transitional zone between the appropriate zone and the inappropriate zone.

The potentially suitable areas for 50 peatland plants in Sichuan Province vary depending on different future scenarios (**Figure 7**, **Table 3**). At the end of the 2100s: In the SSP126 scenario, the area of the low suitability zone increases to 8.33×10^4^ km², while the area of the medium suitability zone and high suitability zone decreases to 3.41×10^4^ km² and 3.27×10^4^ km², respectively; In the SSP370 scenario, the low suitability zone expanded to 9.75×10^4^ km², while the medium suitability zone and the high suitability zone decreased to 2.71 ×10^4^ km² and 2.89×10^4^ km², respectively; In the SSP585 scenario, the low and medium suitability zones increased to 8.55×10^4^ km² and 3.74×10^4^ km², respectively, while only the high suitability zone shrank to 3.50×10^4^ km².

**Table 3:** Potential adaptation area and changes under different scenarios (×10^4^ km^2^).

Scenario		Low Suit	Change (%)	Middle Suit	Change (%)	High Suit	Change (%)	Total Suit	Change (%)
Current		8.00	0	3.45	0	3.71	0	15.16	0
2071-2100	SSP126	8.33	4.12	3.41	-1.16	3.27	-11.86	15.01	-0.99
	SSP370	9.75	21.88	2.71	-21.44	2.89	-22.10	15.35	1.25
	SSP585	8.55	6.88	3.74	8.40	3.50	-5.66	15.79	4.16

### 3.5 Peatland plant centroid shifts in Sichuan under different scenarios

**Figure 8:**
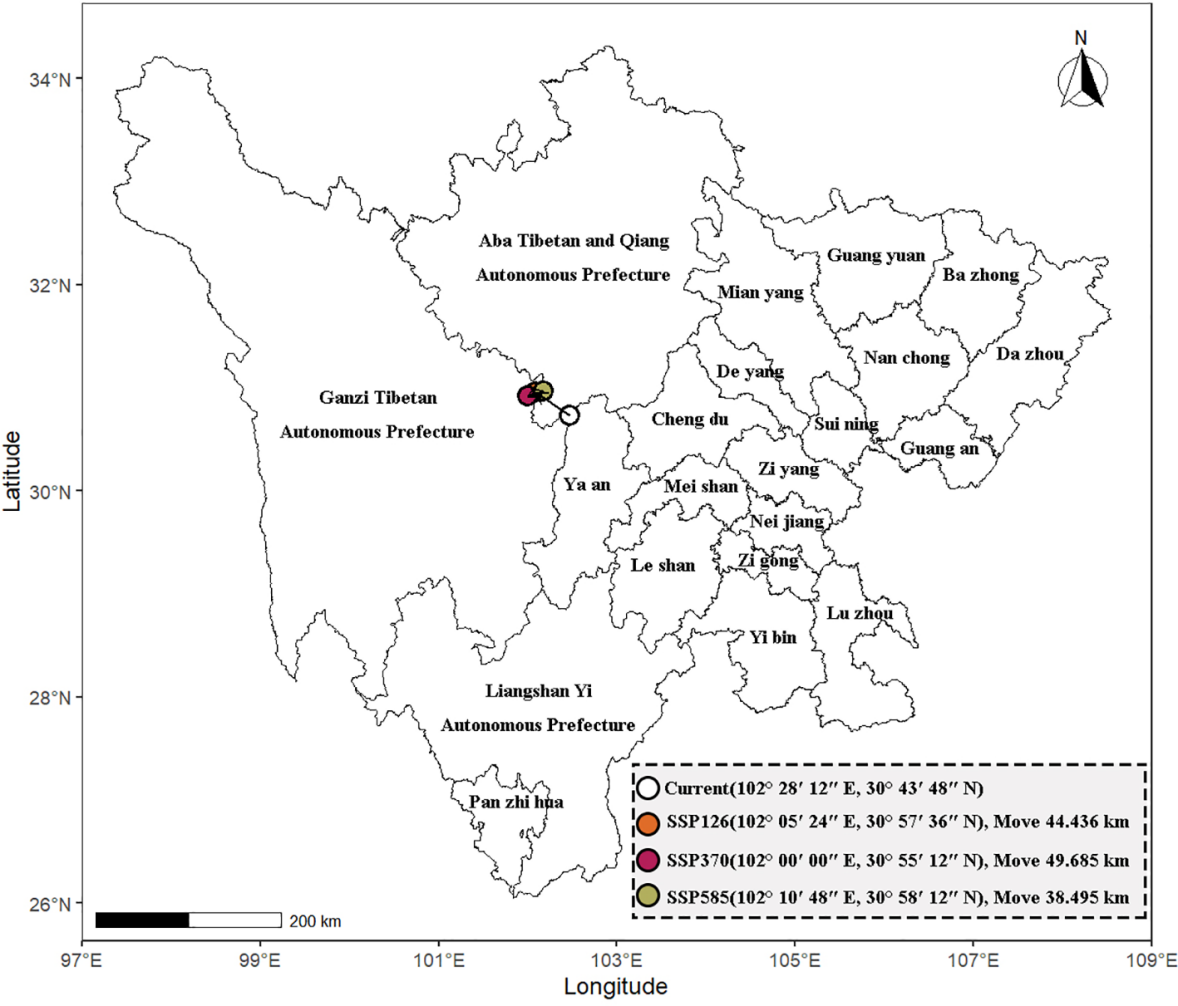
Center of mass offset in different scenarios.

The distribution centroid of peatland plants in Sichuan Province shifted northward under three different climate scenarios (**Figure 8**). From 1981 to 2010, the distribution centroid was roughly located in the northwest of Baoxing County, Ya’an (102°28′12″E, 30°43′48″N). By the end of the 2100s, in the context of SSP126, it is expected that the distribution center of mass will shift in a northwest direction by 44.436 km to the vicinity of San Village, La Yue Mountain, Danba County, Ganzi Prefecture (102°05′24″E, 30°57′36″N). In the context of SSP370, the distribution centroid is estimated to move 49.685 km northwest to the vicinity of Bake Village in Danba County, Ganzi Prefecture (102°00′00″E, 30°55′12″N). In the context of SSP585, the distribution centroid is shifted northwestward by 38.495 km to Xiaojin County, Aba Prefecture (102°10′48″E, 30°58′12″N).

## 4 Discussion

### 4.1 Analysis of factors influencing plant distribution in advantageous peatlands in Sichuan province

The analysis identifies SCD, Slope, TWI, Bio15, and Bio3 as key factors influencing plant distribution in Sichuan’s peatlands.

The optimal range for SCD is between 120 and 240 days, with the highest occurrence rate at 180 days. This reflects the higher tolerance of peatland plants in Sichuan Province to snow cover, making them more suitable for medium-to-long snow cover periods. Snow functions not just as a thermal buffer, attenuating ground temperature fluctuations and maintaining generally stable thermal conditions, thereby insulating vegetation from harm caused by extreme cold or rapid temperature shifts (Phoenix, 2018; Kersebaum, 2022). Additionally, snow plays an integral role in hydrological regulation (Zhu et al., 2022; Li et al., 2025). Appropriate snow cover days can not only protect plants from severe cold damage, maintain soil moisture, and promote plant growth and nutrient accumulation, but also provide a better growth season for plants. This helps to enhance the competitive advantage of peatland plants in Sichuan Province, avoid competition with early spring species, and further promote their growth and reproduction under complex climatic conditions (Zhu et al., 2019). Extended snow cover can delay the growing season, leading to unstable water resources, reducing light and warmth time, and ultimately limiting species adaptability (Callaghan and Johansson, 2021). Short snow cover duration can shorten the growing season, increase the risk of freezing damage, and lead to unstable water and nutrient supply (Han et al., 2021). From the perspective of slope, peatland plants in Sichuan Province are highly dependent on flat or slightly gentle slopes and are extremely sensitive to changes in terrain slope. Steep terrain significantly inhibits their survival. Moderate slopes help retain water and maintain soil stability, creating favorable conditions for plant growth and establishment (Li et al., 2020; Qiu et al., 2024; Jourgholami et al., 2020). In contrast, steep slopes increase hydrological loss and soil erosion, limiting plant colonization (Chen et al., 2022; Deng et al., 2025). Bio15 exhibits an ideal range of 600–900 mm, with the highest presence at 800 mm, reflecting that peatland plants in Sichuan Province are more inclined towards moderate precipitation seasonality. Seasonal precipitation provides a steady supply of water, which improves plant water-use efficiency and promotes ideal growth (Liu et al., 2023; Júnior et al., 2023). Both too low and too high Bio15 can disrupt the adapted precipitation rhythm and peatland hydrological balance of the plants, leading to phenological disorders (Zhang et al., 2025). The optimal range of Bio3 is between 3.3 °C and 4.2 °C, indicating that moderate temperature is more suitable for the growth of peatland plants in Sichuan Province. Finally, appropriate isothermal conditions can maintain stable thermal conditions and alleviate heat stress, thereby promoting plant growth, which helps establish species in peatland habitats (Li et al., 2020; Tian et al., 2023). If Bio3 is too low, it will cause extreme temperature stress and intensify freezing and thawing of peat layers, while if it is too high, it will disrupt temperature signals and ecological cycles, both of which will damage the survival conditions of the plants (Gallou et al., 2023; Borbhuyan et al., 2025).

Under various future climatic scenarios, variations in environmental variables such as temperature, moisture, and precipitation significantly influence the distribution pattern of peatland plants in Sichuan Province. These results are consistent with the conclusions drawn in previous studies on the main influencing factors of plant distribution in the Qinghai-Tibet Plateau, Zoige high-altitude peatlands, and western Sichuan (Jun et al., 2024). The ecological niche of Sichuan’s peatland plants is particularly vulnerable to climate change, as these findings demonstrate. Therefore, it is essential to enhance dynamic monitoring and ecological response research on these factors in the context of future climate change.

### 4.2 Analysis of the decline in species richness in peatlands

The MaxEnt model predicts that the distribution centroid of peatland plants in Sichuan Province will shift northward in the future. This is mainly due to the rising temperatures and altered precipitation patterns resulting from climate change, leading to environmental degradation of their original habitats. Climate warming is rendering peatland plants unable to persist in their original habitats, and the future northern regions may become particularly humid, supporting the survival of plants initially adapted to colder climates and thereby facilitating the establishment of habitats for these peatland plants (Zhang et al., 2022; Wang et al., 2024).

At the same time, environmental degradation caused by climate change, along with the loss of ecosystems and ecological niches, has facilitated more invasive alien species in the region (Gong et al., 2020; Siddiqui et al., 2021). Species that fail to successfully migrate may face extinction due to their inability to adapt to their original environment, or because of habitat fragmentation and competition during migration (Wallingford et al., 2020; Duenas et al., 2021; Jones et al., 2021). In turn, successfully migrating species may also disrupt the new ecological balance, exerting competitive pressure on some species in the new community, leading to further loss of species richness (Teem et al., 2020; Gallardo et al., 2024).

In addition, we identified hotspots of species richness and threatened hotspots, and based on this, we overlaid and analyzed the coverage of existing nature reserves (**Supplementary Figures 3–6**). The threatened areas are those where the species richness declines by 5 or more, mainly distributed in the northwest of Shiqu County, Garze Prefecture, the northeast of Aba Prefecture, and the Minshan Qionglai Mountains. These regions have a large altitude span, complex terrain, and substantial variation in resource availability, which means that the survival of peatland plants in these areas will be significantly challenged, resulting in a notable decline in species diversity (Xiao et al., 2023; Dong et al., 2025). Although several nature reserves have been established in these areas, the current protected area system exhibits uneven distribution and ecological fragmentation. To cope with the northward migration of species and the decrease in species richness, ecological corridors can be built in Pingwu County, Songpan County, Mao County, Wenchuan County, Shiqu County, Dege County, Seda County, Ruoergai County, Qingchuan County, and Beichuan County. These 10 counties are all critical pathways for species’ northward migration and are also core threatened areas under future climate scenarios.

According to the simulation results provided by the National Climate Center of CMA (2022) and the IPCC Sixth Assessment Report (2021), by the 2100s, Sichuan Province will experience warming and precipitation increase in all three scenarios mentioned above (Yazdandoost et al., 2021). Specifically, under the low-emission pathway (SSP126), projections indicate that Sichuan’s mean annual temperature may increase by approximately 1.5 °C to 1.8 °C, accompanied by an estimated 5% rise in total yearly precipitation. Under the medium to high emission route (SSP370), temperature will likely rise by around 3.5 °C and moisture by roughly 10%. The average yearly temperature rises by 5.0 °C, and the precipitation increases by 15% to 20% in the high forcing scenario route (SSP585). The magnitude and uncertainty of future climate change have greatly increased (Xiang et al., 2021). Regionally, the western Sichuan Plateau has seen a far greater rise in temperature than the Sichuan Basin, and the rise in precipitation has mostly occurred at the edges of the plateau and mountainous regions. Under all scenarios, the frequency and intensity of extreme precipitation events are increasing. Such pronounced climate changes exert strong impacts on peatland species richness. Therefore, protecting and restoring habitats for peatland plants in degraded areas of Sichuan Province is particularly urgent.

### 4.3 Research limitations

Overfitting is a common challenge in MaxEnt modeling, especially when sample sizes are limited or feature spaces are high-dimensional, both of which can significantly reduce predictive performance. In addition, the model relies mainly on environmental factors while ignoring key biological interactions such as interspecific competition and predation. This dependence on static environmental conditions also limits the ability to capture dynamic ecological processes, including long-term adaptation and ecosystem change.

## 5 Conclusion

This study applied MaxEnt to model the current distribution of 50 major peatland plants in Sichuan Province and predicted changes in their potential habitat suitability under three different climate scenarios by the end of the 2100s. We identified hotspots for species richness and threatened areas and then overlaid and analyzed the coverage of existing nature reserves. The findings show that during future climate change, the distribution of 50 dominant peatland plants in Sichuan Province is mainly influenced by environmental factors such as SCD, slope, TWI, Bio15, and Bio3. Their potential distribution areas generally shrink, with the most significant decline observed under the SSP370 scenario. The overall species richness has decreased, especially in the plateau and basin edge areas, where the changes in species diversity are most severe, while the stable regions of species diversity are concentrated in Aba Prefecture and Ganzi Prefecture. At the same time, climate change has led to the gradual migration of suitable habitats for 50 peatland plants in Sichuan Province to high latitude regions, with the most significant migration to the northwest under the SSP370 scenario. Finally, it was revealed that under three future climate scenarios, there are still a large number of regions with high species diversity and significant threats from climate change that are not covered by existing natural conservation systems. When building and improving future protection networks, it is necessary to enhance the quality of construction and management of local-level (provincial, municipal, county) protected areas and form a complementary and collaborative mechanism with national-level protected areas.

## Data Availability

The data analyzed in this study is subject to the following licenses/restrictions: The datasets analyzed in this study were obtained through official application from National Earth System Science Data Center, National Science & Technology Infrastructure of China (http://www.geodata.cn)”. and are subject to access restrictions (e.g., non-public distribution, requires formal application to the data provider for legitimate research purposes). Requests to access these datasets should be directed to National Earth System Science Data Center, National Science & Technology Infrastructure of China (http://www.geodata.cn).

## Supplementary material

Supplementary Figure 1 Optimized Maxent model parameters.

Supplementary Figure 2 Sensitivity Analysis of Thresholds for Suitable Habitat.

Supplementary Figure 3 Current Status of Annual Rich Hotspots in Sichuan Province.

Supplementary Figure 4 Threatened high-value ecological and protected areas in Sichuan under the SSP1-2.6 scenario in the mid to late 21st century.

Supplementary Figure 5 Threatened high-value ecological and protected areas in Sichuan under the SSP3-7.0 scenario in the mid to late 21st century.

Supplementary Figure 6 Threatened high-value ecological and protected areas in Sichuan under the SSP5-8.5 scenario in the mid to late 21st century.

Supplementary Table 1 50 dominant peatland plants in Sichuan Province.

Supplementary Table 2 Correlation Matrix of 28 Environmental Variables (When the correlation between two factors is greater than 0.75, only the factor with higher importance is taken).

Supplementary Table 3 Richness area and changes under different scenarios.
