# Supplementary material for: The impact of climate change on plant diversity in peatlands in Sichuan province, China
Source: Front Plant Sci. 2026 Apr 17;17:1762128. doi: 10.3389/fpls.2026.1762128 (PMC13133065; doi:10.3389/fpls.2026.1762128)
Supplement: Supplementary Figure 1 — Optimized Maxent model parameters. [file DataSheet1.pdf]

Supplementary Material

1 Supplementary Figures and Tables

1.1 Supplementary Figures

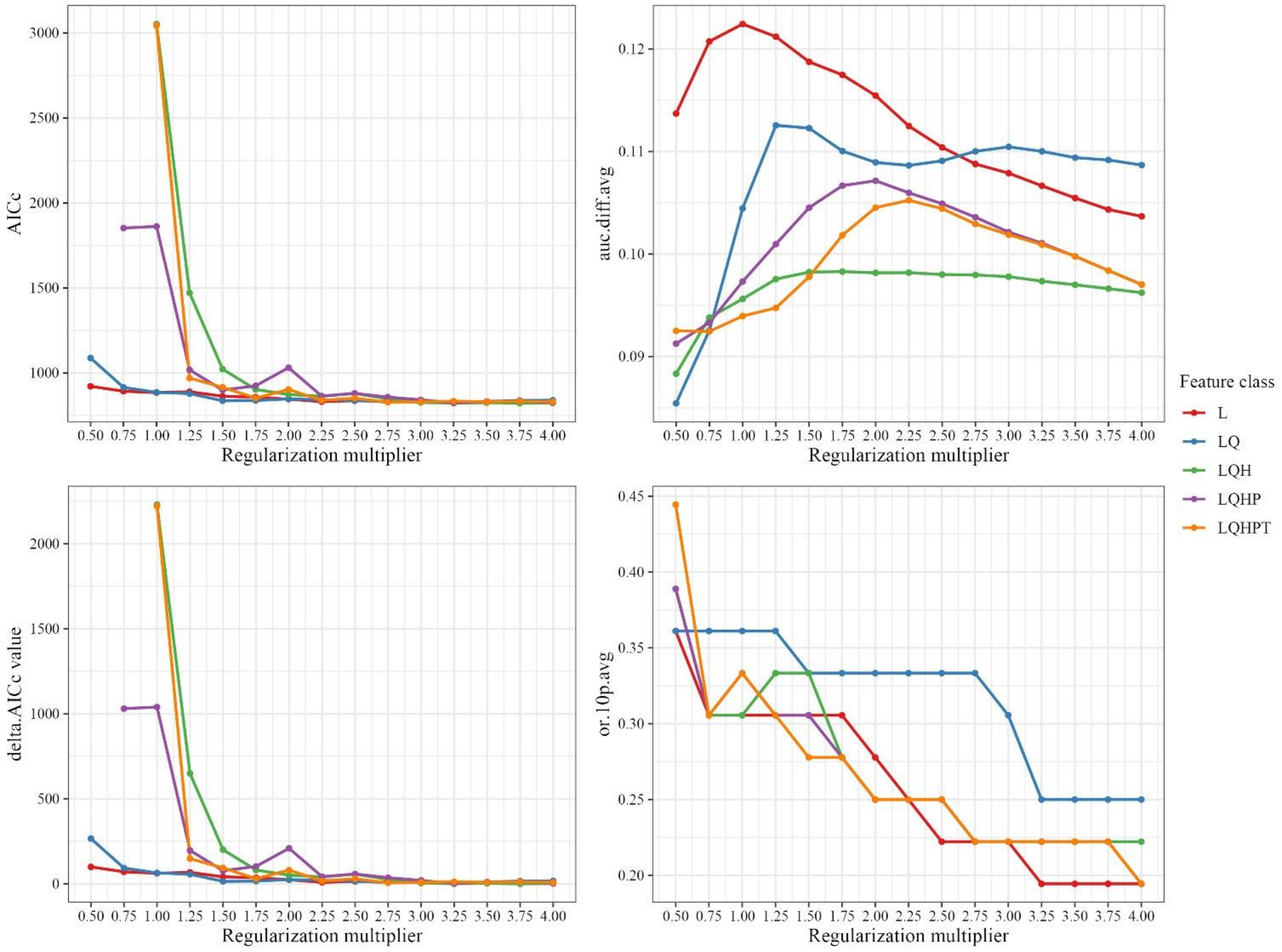

Figure S1 Optimized Maxent model parameters.

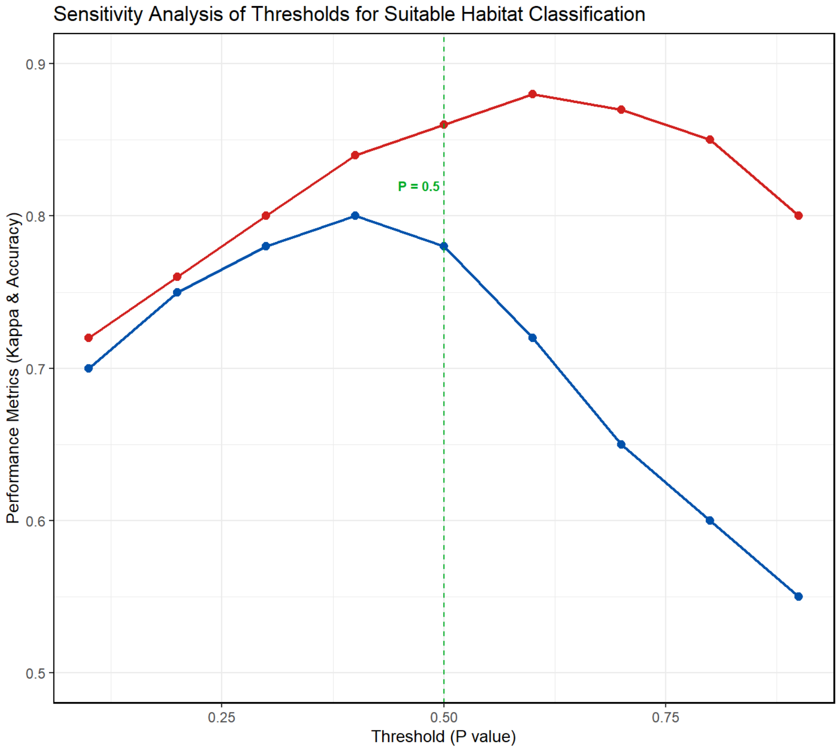

Figure S2 Sensitivity Analysis of Thresholds for Suitable Habitat

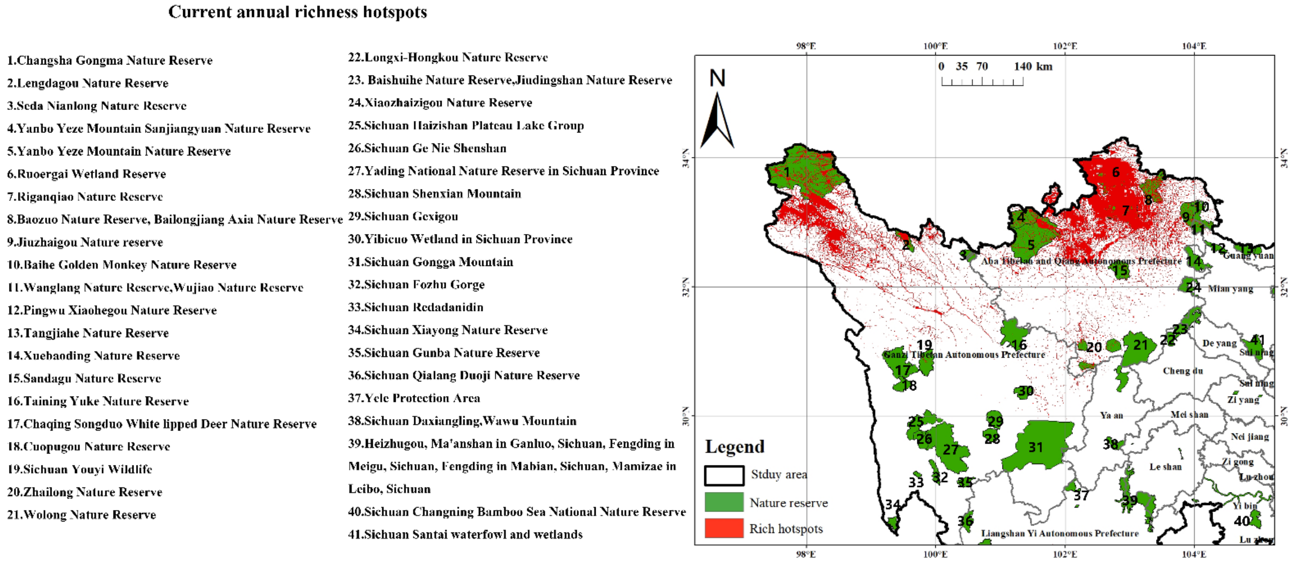

Figure S3 Current Status of Annual Rich Hotspots in Sichuan Province.

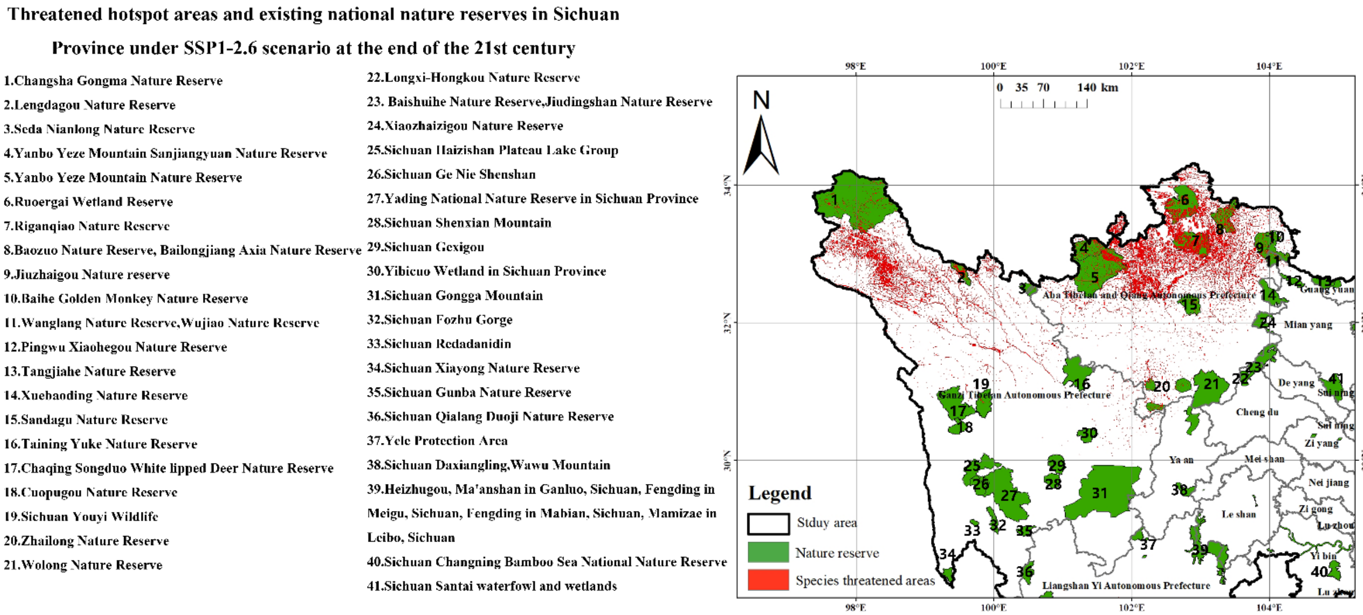

Figure S4 Threatened high-value ecological and protected areas in Sichuan under the SSP1-2.6 scenario at the end of the 21st century.

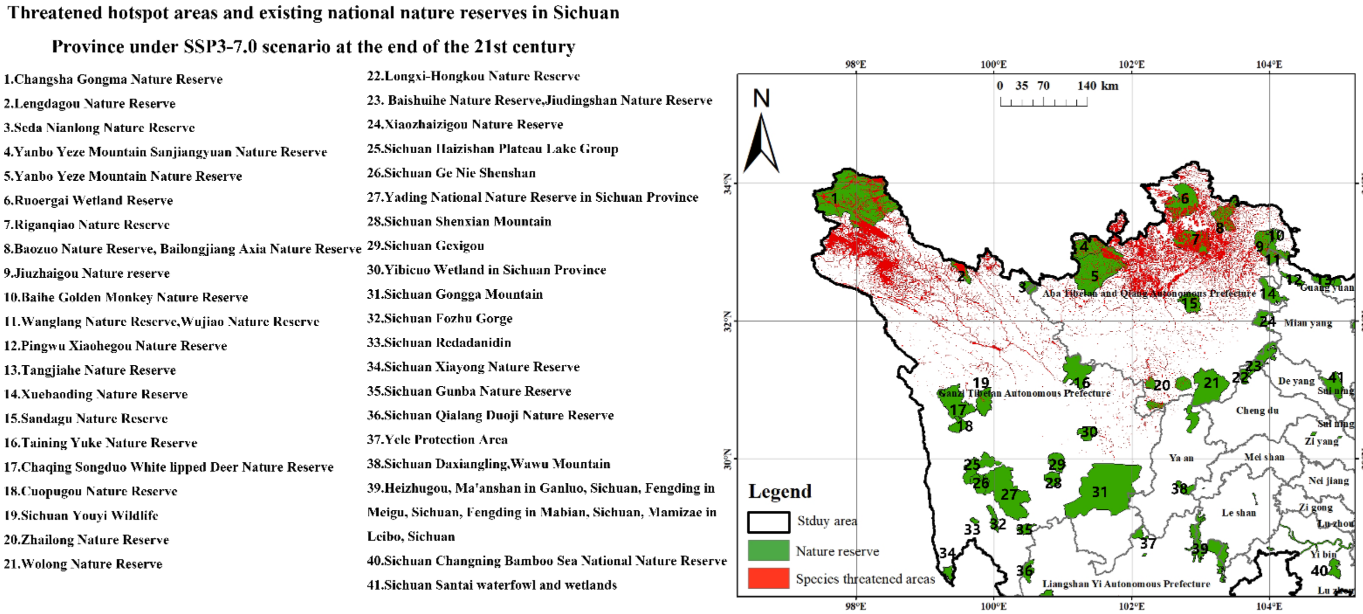

Figure S5 Threatened high-value ecological and protected areas in Sichuan under the SSP3-7.0 scenario at the end of the 21st century.

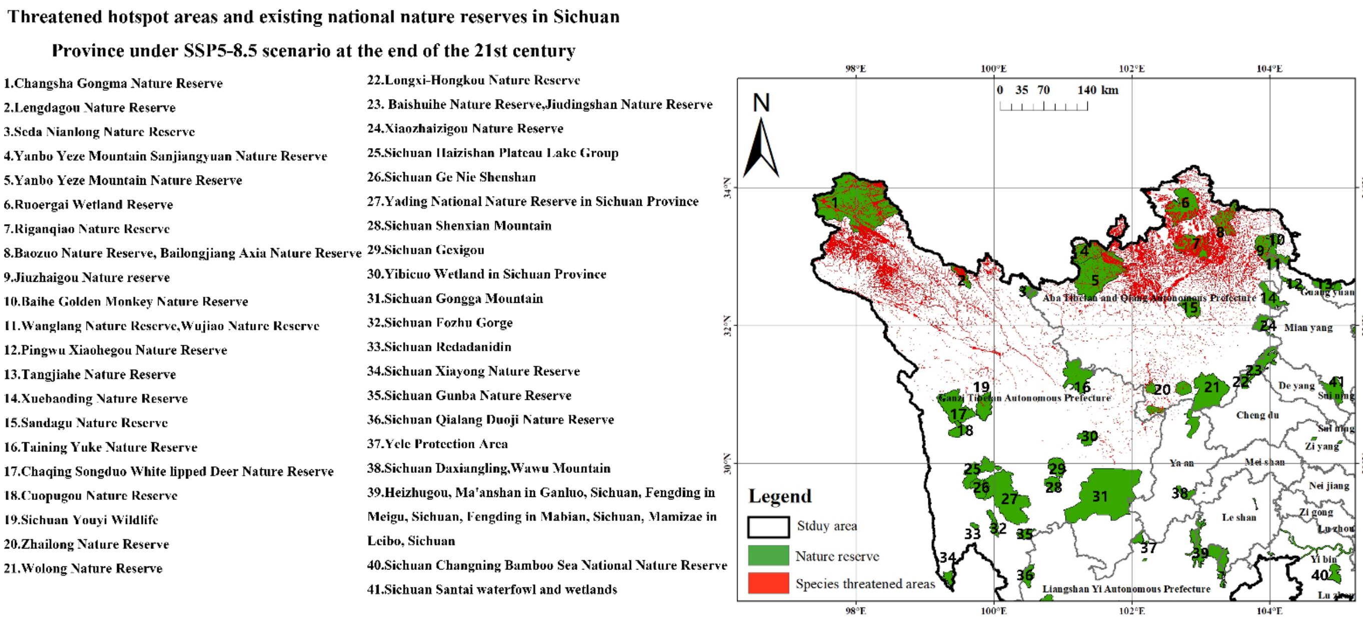

Figure S6 Threatened high-value ecological and protected areas in Sichuan under the SSP5-8.5 scenario at the end of the 21st century.

1.2 Supplementary Tables

Table S1 50 dominant peatland plants in Sichuan Province

Code	Latin names for species	Latin names for families	Latin names for genera	Number of species
**S1**	***Caltha scapose***	***Ranunculaceae***	***Caltha***	
**S2**	***Sanguisorba parviflora***	***Rosaceae***	***Sanguisorba***	
**S3**	***Poa pratensis***	***Poaceae***	***Poa***	
**S4**	***Carex muliensis***	***Cyperaceae***	***Carex***	
**S5**	***Primula secundiflora***	***Primulaceae***	***Primula.***	
**S6**	***Carex parvula***	***Cyperaceae***	***Carex***	

**S7**	***Equisetum hyemale***	***Equisetaceae***	***Equisetum***
**S8**	***Cremanthodium lineare***	***Asteraceae***	***Cremanthodium***
**S9**	***Gentiana lawrencei***	***Gentianaceae***	***Gentiana***
**S10**	***Gentiana straminea***	***Gentianaceae***	***Gentiana***
**S11**	***Utricularia vulgaris***	***Lentibulariaceae***	***Utricularia***
**S12**	***Chamaesium paradoxum***	***Apiaceae***	***Chamaesium***
**S13**	***Oenanthe hookeri***	***Apiaceae***	***Oenanthe***
**S14**	***Juncus allioides***	***Juncaceae***	***Juncus***
**S15**	***Carex brunnescens***	***Cyperaceae***	***Carex***
**S16**	***Juncus tanguticus***	***Juncaceae***	***Juncus***
**S17**	***Poa crymophila***	***Poaceae***	***Poa***
**S18**	***Saussurea stella***	***Asteraceae***	***Saussurea***
**S19**	***Carex setschwanensis***	***Cyperaceae***	***Carex***
**S20**	***Polygonum viviparum***	***Polygonaceae***	***Polygonum***
**S21**	***Leontopodium souliei***	***Asteraceae***	***Leontopodium***
**S22**	***Carex tibetikobresia***	***Cyperaceae***	***Carex***
**S23**	***Carex moorcroftii***	***Cyperaceae***	***Carex***
**S24**	***Carex capillifolia***	***Cyperaceae***	***Carex***

**S25**	***Potentilla potaninii***	***Rosaceae***	***Potentilla***
**S26**	***Pedicularis longiflora***	***Orobanchaceae***	***Pedicularis***
**S27**	***Carex coninux***	***Cyperaceae***	***Carex***
**S28**	***Pedicularis siphonantha***	***Orobanchaceae***	***Pedicularis***
**S29**	***Carex atrofuscoides***	***Cyperaceae***	***Carex***
**S30**	***Ranunculus nephelogenes***	***Ranunculaceae***	***Ranunculus***
**S31**	***Juncus concinnus***	***Juncaceae***	***Juncus***
**S32**	***Ranunculus tanguticus***	***Ranunculaceae***	***Ranunculus***
**S33**	***Bistorta macrophylla***	***Polygonaceae***	***Bistorta***
**S34**	***Triglochin palustris***	***Juncaginaceae***	***Triglochin***
**S35**	***Aster souliei***	***Asteraceae***	***Aster***
**S36**	***Ligularia virgaurea***	***Asteraceae***	***Ligularia***
**S37**	***Plantago depressa***	***Plantaginaceae***	***Plantago***
**S38**	***Triglochin maritima***	***Juncaginaceae***	***Triglochin***
**S39**	***Carex pseuduncinoides***	***Cyperaceae***	***Carex***
**S40**	***Blysmus sinocompressus***	***Cyperaceae***	***Blysmus***
**S41**	***Aster batangensis***	***Asteraceae***	***Aster***
**S42**	***Carex alatauensis***	***Cyperaceae***	***Carex***

**S43**	***Hippuris vulgaris***	***Plantaginaceae***	***Hippuris***
**S44**	***Ranunculus bungei***	***Ranunculaceae***	***Ranunculus***
**S45**	***Chamaesium thalictrifolium***	***Apiaceae***	***Chamaesium***
**S46**	***Lomatogonium macranthum***	***Gentianaceae***	***Lomatogonium***
**S47**	***Ranunculus trichophyllus***	***Ranunculaceae***	***Ranunculus***
**S48**	***Argentina anserina***	***Rosaceae***	***Argentina***
**S49**	***Saxifraga hirculus***	***Saxifragaceae***	***Saxifraga***
**S50**	***Carex parva Nees***	***Cyperaceae***	***Carex***

Table S2 Correlation Matrix of 28 Environmental Variables (When the correlation between two factors is greater than 0.75, only the factor with higher importance is taken)

	Aspect	BD	Bio1	Bio10	Bio11	Bio12	Bio13	Bio14	Bio15	Bio16	Bio17	BIO18	BIO19	Bio2	Bio3	Bio4	Bio5	Bio6	Bio7	Bio8	Bio9	DEM	OC	PH	SCD	Slope	TN	TWI
**Aspect**	1.00	0.03	0.02	0.02	0.02	-0.07	-0.07	-0.01	-0.02	-0.08	-0.02	-0.08	-0.02	0.00	0.01	-0.01	0.02	0.02	-0.01	0.02	0.02	0.00	-0.01	0.00	-0.02	-0.01	-0.01	0.00
**BD**	0.03	1.00	0.90	0.92	0.88	0.06	0.07	0.49	-0.28	0.01	0.47	-0.02	0.47	-0.73	-0.42	-0.48	0.92	0.88	-0.68	0.92	0.88	-0.91	-0.84	0.22	-0.87	-0.34	-0.87	0.39
**Bio1**	0.02	0.90	1.00	0.98	0.99	0.14	0.17	0.56	-0.24	0.10	0.52	0.08	0.52	-0.82	-0.40	-0.66	0.97	0.99	-0.84	0.98	0.99	-0.97	-0.87	0.07	-0.97	-0.27	-0.91	0.37
**Bio10**	0.02	0.92	0.98	1.00	0.96	0.13	0.14	0.60	-0.32	0.06	0.57	0.04	0.57	-0.82	-0.51	-0.52	1.00	0.96	-0.75	1.00	0.96	-0.99	-0.88	0.14	-0.94	-0.33	-0.92	0.40
**Bio11**	0.02	0.88	0.99	0.96	1.00	0.15	0.20	0.52	-0.18	0.14	0.48	0.11	0.48	-0.82	-0.32	-0.75	0.94	0.99	-0.89	0.96	1.00	-0.93	-0.85	0.02	-0.97	-0.22	-0.90	0.34
**Bio12**	-0.07	0.06	0.14	0.13	0.15	1.00	0.87	0.44	-0.30	0.92	0.53	0.92	0.52	-0.44	-0.40	-0.18	0.09	0.19	-0.29	0.12	0.14	-0.17	-0.03	-0.54	-0.14	0.11	-0.06	-0.19
**Bio13**	-0.07	0.07	0.17	0.14	0.20	0.87	1.00	0.21	0.14	0.97	0.28	0.97	0.27	-0.42	-0.26	-0.31	0.10	0.24	-0.38	0.13	0.20	-0.16	-0.07	-0.50	-0.20	0.09	-0.10	-0.19
**Bio14**	-0.01	0.49	0.56	0.60	0.52	0.44	0.21	1.00	-0.70	0.23	0.98	0.23	0.98	-0.67	-0.65	-0.17	0.60	0.57	-0.43	0.61	0.52	-0.62	-0.50	-0.19	-0.50	-0.25	-0.53	0.20
**Bio15**	-0.02	-0.28	-0.24	-0.32	-0.18	-0.30	0.14	-0.70	1.00	0.08	-0.74	0.07	-0.74	0.32	0.54	-0.18	-0.34	-0.21	0.02	-0.31	-0.18	0.37	0.22	0.03	0.18	0.12	0.23	-0.14
**Bio16**	-0.08	0.01	0.10	0.06	0.14	0.92	0.97	0.23	0.08	1.00	0.30	0.99	0.29	-0.38	-0.24	-0.28	0.03	0.17	-0.34	0.07	0.13	-0.09	0.00	-0.54	-0.13	0.12	-0.03	-0.21
**Bio17**	-0.02	0.47	0.52	0.57	0.48	0.53	0.28	0.98	-0.74	0.30	1.00	0.30	1.00	-0.68	-0.72	-0.12	0.57	0.54	-0.40	0.57	0.48	-0.61	-0.47	-0.20	-0.46	-0.22	-0.49	0.16
**BIO18**	-0.08	-0.02	0.08	0.04	0.11	0.92	0.97	0.23	0.07	0.99	0.30	1.00	0.29	-0.36	-0.24	-0.26	0.00	0.15	-0.32	0.04	0.11	-0.07	0.02	-0.55	-0.11	0.12	-0.01	-0.21
**BIO19**	-0.02	0.47	0.52	0.57	0.48	0.52	0.27	0.98	-0.74	0.29	1.00	0.29	1.00	-0.68	-0.70	-0.12	0.57	0.54	-0.40	0.57	0.48	-0.60	-0.47	-0.20	-0.46	-0.23	-0.49	0.16
**Bio2**	0.00	-0.73	-0.82	-0.82	-0.82	-0.44	-0.42	-0.67	0.32	-0.38	-0.68	-0.36	-0.68	1.00	0.70	0.55	-0.79	-0.87	0.83	-0.82	-0.82	0.84	0.72	0.16	0.80	0.16	0.77	-0.21
**Bio3**	0.01	-0.42	-0.40	-0.51	-0.32	-0.40	-0.26	-0.65	0.54	-0.24	-0.72	-0.24	-0.70	0.70	1.00	-0.18	-0.51	-0.40	0.19	-0.50	-0.32	0.56	0.41	-0.03	0.33	0.24	0.42	-0.17
**Bio4**	-0.01	-0.48	-0.66	-0.52	-0.75	-0.18	-0.31	-0.17	-0.18	-0.28	-0.12	-0.26	-0.12	0.55	-0.18	1.00	-0.48	-0.72	0.91	-0.53	-0.74	0.49	0.48	0.27	0.71	-0.11	0.53	-0.08
**Bio5**	0.02	0.92	0.97	1.00	0.94	0.09	0.10	0.60	-0.34	0.03	0.57	0.00	0.57	-0.79	-0.51	-0.48	1.00	0.94	-0.71	1.00	0.94	-0.98	-0.87	0.17	-0.92	-0.36	-0.91	0.42
**Bio6**	0.02	0.88	0.99	0.96	0.99	0.19	0.24	0.57	-0.21	0.17	0.54	0.15	0.54	-0.87	-0.40	-0.72	0.94	1.00	-0.90	0.96	0.99	-0.95	-0.86	-0.01	-0.97	-0.24	-0.90	0.33
**Bio7**	-0.01	-0.68	-0.84	-0.75	-0.89	-0.29	-0.38	-0.43	0.02	-0.34	-0.40	-0.32	-0.40	0.83	0.19	0.91	-0.71	-0.90	1.00	-0.75	-0.89	0.73	0.69	0.24	0.85	0.05	0.73	-0.17
**Bio8**	0.02	0.92	0.98	1.00	0.96	0.12	0.13	0.61	-0.31	0.07	0.57	0.04	0.57	-0.82	-0.50	-0.53	1.00	0.96	-0.75	1.00	0.96	-0.98	-0.88	0.13	-0.94	-0.33	-0.92	0.40
**Bio9**	0.02	0.88	0.99	0.96	1.00	0.14	0.20	0.52	-0.18	0.13	0.48	0.11	0.48	-0.82	-0.32	-0.74	0.94	0.99	-0.89	0.96	1.00	-0.94	-0.85	0.02	-0.97	-0.23	-0.90	0.34
**DEM**	0.00	-0.91	-0.97	-0.99	-0.93	-0.17	-0.16	-0.62	0.37	-0.09	-0.61	-0.07	-0.60	0.84	0.56	0.49	-0.98	-0.95	0.73	-0.98	-0.94	1.00	0.86	-0.13	0.93	0.32	0.90	-0.41
**OC**	-0.01	-0.84	-0.87	-0.88	-0.85	-0.03	-0.07	-0.50	0.22	0.00	-0.47	0.02	-0.47	0.72	0.41	0.48	-0.87	-0.86	0.69	-0.88	-0.85	0.86	1.00	-0.20	0.83	0.30	0.96	-0.32
**PH**	0.00	0.22	0.07	0.14	0.02	-0.54	-0.50	-0.19	0.03	-0.54	-0.20	-0.55	-0.20	0.16	-0.03	0.27	0.17	-0.01	0.24	0.13	0.02	-0.13	-0.20	1.00	0.01	-0.29	-0.20	0.33
**SCD**	-0.02	-0.87	-0.97	-0.94	-0.97	-0.14	-0.20	-0.50	0.18	-0.13	-0.46	-0.11	-0.46	0.80	0.33	0.71	-0.92	-0.97	0.85	-0.94	-0.97	0.93	0.83	0.01	1.00	0.19	0.88	-0.30
**Slope**	-0.01	-0.34	-0.27	-0.33	-0.22	0.11	0.09	-0.25	0.12	0.12	-0.22	0.12	-0.23	0.16	0.24	-0.11	-0.36	-0.24	0.05	-0.33	-0.23	0.32	0.30	-0.29	0.19	1.00	0.31	-0.57
**TN**	-0.01	-0.87	-0.91	-0.92	-0.90	-0.06	-0.10	-0.53	0.23	-0.03	-0.49	-0.01	-0.49	0.77	0.42	0.53	-0.91	-0.90	0.73	-0.92	-0.90	0.90	0.96	-0.20	0.88	0.31	1.00	-0.35
**TWI**	0.00	0.39	0.37	0.40	0.34	-0.19	-0.19	0.20	-0.14	-0.21	0.16	-0.21	0.16	-0.21	-0.17	-0.08	0.42	0.33	-0.17	0.40	0.34	-0.41	-0.32	0.33	-0.30	-0.57	-0.35	1.00

Table S3 The predictive performance (AUC value) of the MaxEnt model for simulating the present distribution of dominant peatland plants in Sichuan Province.

Latin names for species	AUC Value
Caltha scapose	**0.9582**
Sanguisorba parviflora	**0.9335**
Poa pratensis	**0.9849**
Carex muliensis	**0.9762**
Primula secundiflora	**0.9094**
Carex parvula	**0.9016**
Equisetum hyemale	**0.9452**
Cremanthodium lineare	**0.9237**
Gentiana lawrencei	**0.9049**
Gentiana straminea	**0.9430**
Utricularia vulgaris	**0.9812**
Chamaesium paradoxum	**0.9296**
Oenanthe hookeri	**0.9085**
Juncus allioides	**0.9563**

Carex brunnescens	**0.9477**
Juncus tanguticus	**0.9147**
Poa crymophila	**0.9253**
Saussurea stella	**0.9148**
Carex setschwanensis	**0.8560**
Polygonum viviparum	**0.9188**
Leontopodium souliei	**0.9322**
Carex tibetikobresia	**0.8997**
Carex moorcroftii	**0.9290**
Carex capillifolia	**0.9267**
Potentilla potaninii	**0.9608**
Pedicularis longiflora	**0.9729**
Carex coninux	**0.8923**
Pedicularis siphonantha	**0.9618**
Carex atrofuscoides	**0.9374**
Ranunculus nephelogenes	**0.9166**

Juncus concinnus	**0.9197**
Ranunculus tanguticus	**0.9321**
Bistorta macrophylla	**0.9151**
Triglochin palustris	**0.9531**
Aster souliei	**0.9261**
Ligularia virgaurea	**0.8977**
Plantago depressa	**0.9099**
Triglochin maritima	**0.9273**
Carex pseuduncinoides	**0.9027**
Blysmus sinocompressus	**0.9887**
Aster batangensis	**0.9100**
Carex alatauensis	**0.9615**
Hippuris vulgaris	**0.9970**
Ranunculus bungei	**0.9920**
Chamaesium thalictrifolium	**0.9030**
Lomatogonium macranthum	**0.8923**
Ranunculus trichophyllus	**0.9570**

Argentina anserina	**0.9650**
Saxifraga hirculus	**0.8566**
Carex parva Nees	**0.8638**

Table S4 Richness area and changes under different scenarios (×10^4 km^2).

Scenario		[5-10)	Change (%)	[10-15)	Change (%)	[15-20)	Change (%)	[20-25)	Change (%)	[25-30)	Change (%)	[30-35)	Change (%)	＞35	Change (%)
Current		8.05	0.00	4.37	0.00	3.98	0.00	3.47	0.00	2.88	0.00	2.14	0.00	2.92	0.00
	SSP126	8.73	8.45	4.53	3.66	2.87	-27.89	1.93	-44.38	1.30	-54.86	0.82	-61.68	1.12	-61.64
2071-2100	SSP370	6.88	-14.53	4.45	1.83	3.19	-19.85	2.20	-36.60	1.31	-54.51	0.77	-63.99	0.97	-66.78
	SSP585	7.08	-12.04	4.19	-4.12	3.00	-24.62	2.13	-38.62	1.38	-52.08	0.88	-58.88	1.13	-61.3
